# PAIRING STORIES AND EDUCATIONAL CONTENT FOR A CULTURALLY CELEBRATORY HEALTHY AGING COMMUNITY RESOURCE

**DOI:** 10.1093/geroni/igz038.1906

**Published:** 2019-11-08

**Authors:** Raina L Croff, Edline Francois, Caroline Lawrence, Zoe Rothberg, Juell Towns, Patrice Fuller, Andre Pruitt, Jeffrey Kaye

**Affiliations:** 1 Oregon Health & Science University, Portland, Oregon, United States; 2 Lewis and Clark College, Portland, Oregon, United States; 3 Portland State University, Portland, Oregon, United States

## Abstract

Culturally celebratory programming exceeds cultural relevancy, engaging participants in celebration-making and culture-creating. African Americans aged 55+ in the Sharing History through Active Reminiscence and Photo-imagery (SHARP) study celebrate their heritage in gentrifying neighborhoods through walking-reminiscence sessions; they create culture through discussing ideas, beliefs, norms, values, and shared experiences of the past while considering these aspects within presently changing cultural dynamics. The SHARP study’s narrative approach supports cognitively healthy behaviors and community priorities of cultural preservation in response to marginalization. The SHARP smartphone application houses 72 themed walking routes in Portland, Oregon’s historically Black neighborhoods. One-mile routes with GPS-triggered historical neighborhood images prompt conversational reminiscence among walking triads. Recorded narratives are organized in a process called storytabling and thematically coded. Selections referencing cognitively healthy behaviors are flagged for pairing with online brain health content tested by 12 African Americans aged 55+. Historical images and narratives anchor educational content to relatable life experiences, framing healthy aging in a culturally celebratory, neighborhood context to improve applicability. The online resource, routes, and recorded narratives are community deliverables. Currently, 254 walking narratives from 2016-2018 walkers (n=40; 8 with mild cognitive impairment) have been transcribed and 60 analyzed. Walkers found image prompts and walking within triads of similar sociocultural backgrounds as highly motivating, healthy ways of addressing change. Content testers found narratives lent depth, meaning, and a sense of cultural resilience to educational content. Narrative approaches situate cognitive health in participant-driven terms and experiences, informing brain health best practices for marginalized and minority populations.

